# Supplementary figures and images for: Development of automatic generation system for lung nodule finding descriptions
Source: PLoS One. 2024 Mar 21;19(3):e0300325. doi: 10.1371/journal.pone.0300325 (PMC10956853; doi:10.1371/journal.pone.0300325)

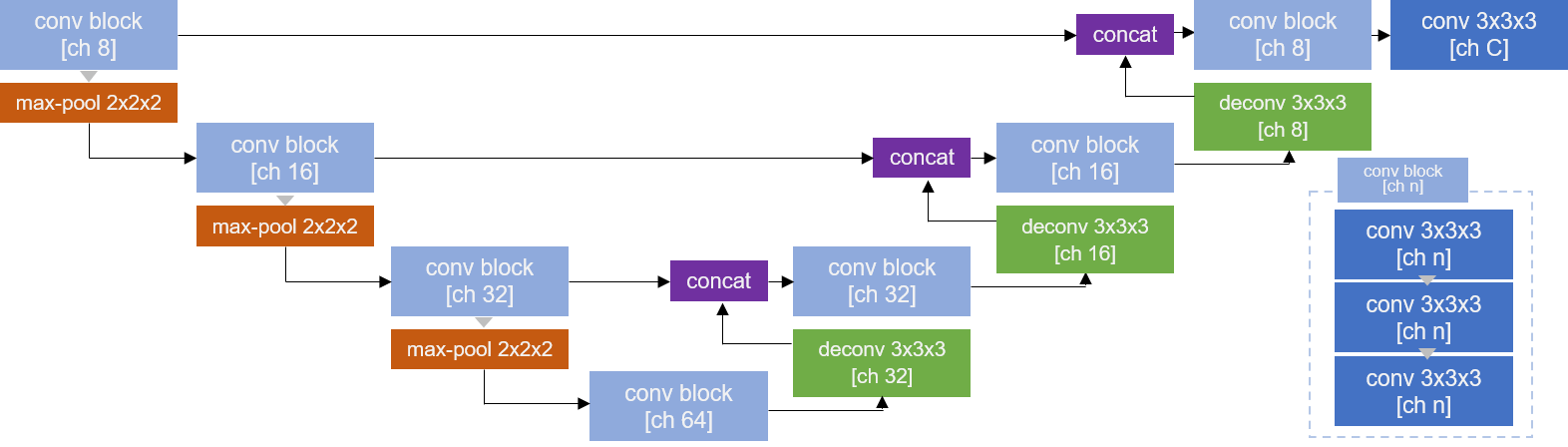

Supplement: S1 Fig — It consists of 22 3D-convolution layers, 3 max pooling layers, and 3 3D-deconvolution layers. Batch normalization layer and ReLU layer following each convolution layer. (TIF) [file pone.0300325.s001.tif]

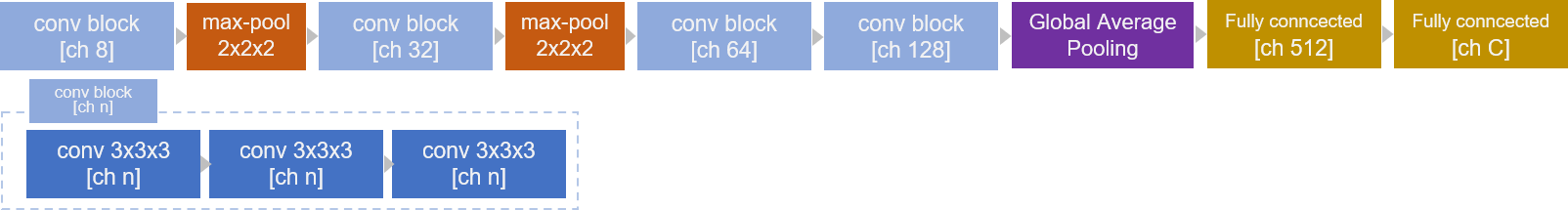

Supplement: S2 Fig — It consists of 12 3D-convolution layers and 2 fully connected layers. The output of the final convolution layer is compressed to 128 dimensions by global average pooling layer. Batch normalization layer and ReLU layer following each convolution layer. C represents the number of classes to be classified, which we set to 16 in our experiment. (TIF) [file pone.0300325.s002.tif]

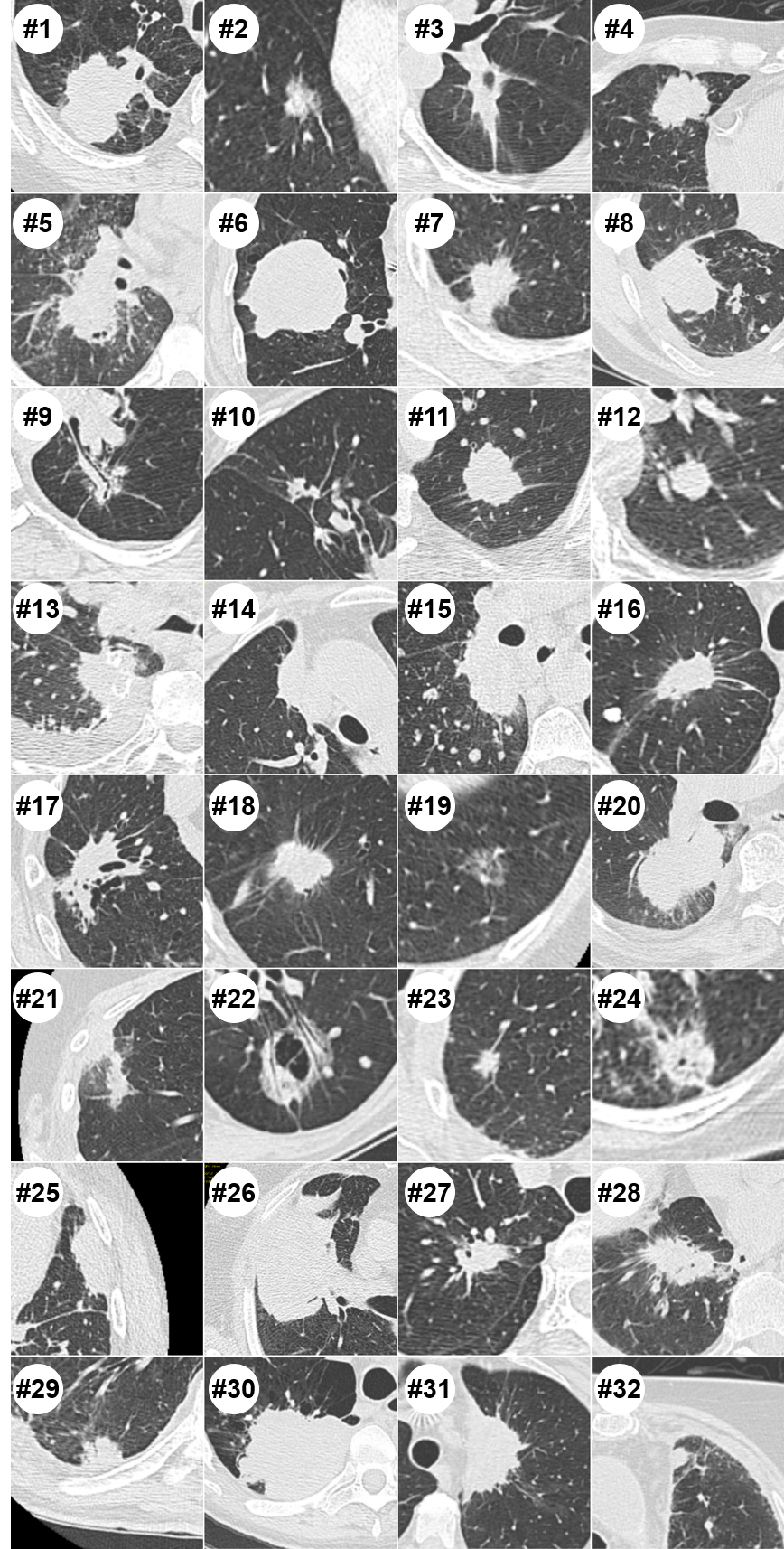

Supplement: S3 Fig — Reprinted from https://www.cancerimagingarchive.net/collection/rider-lung-ct/ under a CC BY license, with permission from Zhao, B., Schwartz, L. H., & Kris, M. G., original copyright 2015. (TIF) [file pone.0300325.s003.tif]
