# Supplementary material for: Development of automatic generation system for lung nodule finding descriptions
Source: PLoS One. 2024 Mar 21;19(3):e0300325. doi: 10.1371/journal.pone.0300325 (PMC10956853; doi:10.1371/journal.pone.0300325)
Supplement: Attachment — Submitted filename: Response_to_Reviewers.docx [file pone.0300325.s005.docx]

Dear Dr. Steve Zimmerman,

Thank you for giving me the opportunity to submit a revised draft of our manuscript titled “Development of automatic generation system for lung nodule finding descriptions” to *PLOS ONE*. We appreciate the time and effort that you and the reviewers have dedicated to providing your valuable feedback on our manuscript. We are grateful to the reviewers for their insightful comments on our manuscript. We have been able to incorporate changes to reflect most of the suggestions provided by the reviewers. We hope that our edits and the responses we provide below satisfactorily address all the issues and concerns you and the reviewers have noted. We have highlighted the changes within the manuscript.

To facilitate your review of our revisions, the following is a point-by-point response to the questions and comments delivered in your letter dated 13-Dec-2023.

**RESPONSE TO JOURNAL**

We have highlighted the changes related to the journal requirements in green in the revised manuscript to provide clarification.

**Comment 1:** Please ensure that your manuscript meets PLOS ONE's style requirements, including those for file naming.

**Response:** Thank you for reviewing our manuscript. We have carefully reviewed PLOS ONE's style requirements, and made the necessary revisions to ensure compliance.

**Comment 2**: When you resubmit, please ensure that you provide the correct grant numbers for the awards you received for your study in the ‘Funding Information’ section.

**Response:** We apologize for not including the grant number in the initial submission. The grant number is “2618Dn-02b”. However, there was no way to correct the Financial Disclosure Statement in the resubmission system, so we have included the change in the Cover Letter for your review.

**Comment 3**: Upon re-submitting your revised manuscript, please upload your study’s minimal underlying data set as either Supporting Information files or to a stable, public repository and include the relevant URLs, DOIs, or accession numbers within your revised cover letter.

**Response:** Thank you for your comment. In response to this, we have added detailed information, such as thumbnail images for identifying the lung nodules used in the Observer Performance Test, radiologist-generated descriptions, gold standards, and statistical data. These additions have been included as S3 Fig and S4 Data in the Supporting Information. These additional files provide the necessary details and data for better understanding and replication of this study. Additionally, there was a slight change in the decimal value of the standard deviation in Table 5 due to the switch from script-based to Excel-based calculation of statistical data for the submission of this data set. However, this difference is minuscule and does not affect our claims. We have ensured consistency by matching the values in the reviced manuscript with those in S4 Data. If you need any further information, please don't hesitate to let us know.

**Comment 4**: Please include your full ethics statement in the ‘Methods’ section of your manuscript file. In your statement, please include the full name of the IRB or ethics committee who approved or waived your study, as well as whether or not you obtained informed written or verbal consent. If consent was waived for your study, please include this information in your statement as well.

**Response:** Thank you for your comments. We have addressed this issue by posting the full ethics statement in the Materials and Methods section of the manuscript file. In this statement, we have included the official name of the ethics committee that approved this study. In addition, we stated that patient consent was waived for our study.

**Comment 5:** We note that Figure(s) 1, 2, 4, 6 and 9 in your submission contain copyrighted images.
**Response:** Thank you for bringing this to our attention. We would like to clarify that for Figures 1, 2, and 6, we have obtained permission from one of the co-authors who owns the images used in those figures. We have uploaded the content permission form for these figures.

Regarding Figures 4 and 9, we would like to inform you that these images are sourced from the RIDER Lung CT dataset, which is published under the CC BY 3.0 license. As per the assessment by the PLOS office, it has been determined that no further permission from the original copyright holders is required for the usage of these images. We can provide the appropriate reference and citation for these figures in accordance with the licensing terms.

We would also like to inform you that as per your instructions, we have added the necessary text to the captions of Figure 1, 2, 4, 6, 9, and S3 Fig.

**Comment 6:** Please review your reference list to ensure that it is complete and correct.

**Response:** We apologize for the error in our reference list. Upon reviewing it again, we noticed a mistake in reference 27. We have corrected the reference and ensured that our reference list is complete and accurate.

**RESPONSE TO REVIEWER 1**

We have highlighted the changes related to the reviewer comments in cyan in the revised manuscript to provide clarification.

**Comment 1:** Which were the inclusion criteria of all the radiology reports (known lung nodules, oncologic history, screening CT, etc.) ?

**Response:** Thank you for your question. We extracted and collected radiology reports that indicated the presence of lung nodules. We did not specifically consider the inclusion criteria such as nodule’s malignancy or the purpose of the examination.

We have added the following sentences in lines 93-96 of the revised manuscript.

*We analyzed the reports to identify examinations in which at least one lung nodule was detected. Based on the results, images and reports were collected without considering the oncological history or examination purpose (screening, preoperative evaluation, etc.).*

**Comment 2:** Regarding the bronchopulmonary segments’ prediction, have the author evaluated the system on patients with previous lung resection, with lesions involving two or more lobes or in cases of pulmonary atelectasis or Azygos lobe?

**Response:** We appreciate your thoughtful comment. The dataset used for evaluation in this study was not specifically collected to assess the performance on cases with previous lung resection, with lesions involving multiple lobes, pulmonary atelectasis, or Azygos lobe. Therefore, we have not adequately evaluated the performance of the system on these types of cases. Upon reviewing the evaluation dataset, we have found one case with partial lung resection and five cases with inflammatory diseases that caused lung deformities. The system achieved an exact match rate of 88.0% for the former case, and a rate of 60.0% for the latter cases.

**Comment 3:** Have the author evaluated the presence of any anatomical features that could improve the match of the report as the presence of complete fissures.

**Response:**

Thank you for your question. Upon additional investigation, we found that there were 21 cases in which the target lung nodules were in the lobe with insufficient interlobar fissures. We examined the exact match rate between the gold standard and prediction results for these cases. The exact match rate was 71.4% in cases with insufficient interlobar fissures and 76.7% in cases without insufficient interlobar fissures (p = 0.15; two-tailed *t*-test).

We have added one paragraph in the ‘Results’ section of the revised manuscript (lines 335-340) based on your Comment 2 & 3.

**Comment** **4:** Have the authors compared the description of the lung nodule with eventual pathological features?

**Response:** Thanks for your question. In this study, we did not compare the description of the lung nodules with pathological features. The RIDER dataset we used is composed of cases with small cell lung cancer, but more detailed pathological features are not publicly available. Comparing the pathological features with the analysis results of the lung nodule characteristics is an area for future work.

We have added the following statements to the ‘Discussion’ section of the revised manuscript (lines 496-499).

*Second, we have not compared the results of nodule classification with the eventual pathological features. In future work, we plan to investigate the impact of this AI system on the differential diagnosis of lung nodules by comparing its output with the pathological features.*

**Comment 5:** In the same way, is it possible evaluate the system by adding the time evolution of the same lung nodule in order to better distinguish benign lesions from the malignant ones?

**Response:** Thank you for your thoughtful comment. We believe it is indeed possible to evaluate the system by incorporating the time evolution of lung nodules. By tracking the changes in characteristics over time using our system, we expect to better distinguish between benign and malignant lesions.

**RESPONSE TO REVIEWER 2**

**Comment 1:** I think the technical descriptions regarding the system construction, or the evaluation may be too complicated for general readers.

Therefore, I strongly request that they should be revised to be more understandable for many readers.

**Response:** We appreciate your comment regarding the complexity of the technical descriptions. We agree that our ’Materials and Methods’ section may be difficult for general readers to understand. Therefore, we have revised the content with the assistance of an editing service to make it more accessible and comprehensible. Specifically, the text structure was changed to improve the clarity and readability of the information. Changes are highlighted by yellow in the revised manuscript.

**Comment 2:** In various deep learning process, on what basis was the sample size determined? In line 262, the experiments were performed 5 times. In line 270, the experiments were performed 7 times. What is the difference between 2 sentences?

**Response:** Thank you for your comment. The required number of trials was determined through a priori power analysis, with an α level of 0.05, a β level of 0.80. We have added clarification in lines 263-264 of the revised manuscript.

**Comment 3:** Other minor points of my concern are as follows.

Line 93 autors→authors
Please correct.

Regarding the slice thickness in table1, the scale is centimeter (cm).
Is it correct?

**Response:** Thank you for your careful review. We have made the necessary correction of "autors" to "authors" in line 97. Additionally, we have revised the scale in Table 1 to reflect the correct unit of measurement, which is mm. Thank you once again for bringing these to our attention.

Again, thank you for giving us the opportunity to strengthen our manuscript with your valuable comments and queries. We have worked hard to incorporate your feedback and hope that these revisions persuade you to accept our submission.

Sincerely,

Yohei Momoki

Fujifilm Corporation

2-26-30, Nishiazabu, Minato-ku, Tokyo, Japan.

mail: [yohei.momoki@fujifilm.com](mailto:yohei.momoki@fujifilm.com)
